# Validation of a Revised V-Slope Algorithm for Automated First Ventilatory Threshold Detection in Healthy Adults

**DOI:** 10.1186/s40798-026-01124-8

**Published:** 2026-10-11

**Authors:** Margot Vermeiren, Kaatje Goetschalckx, Herwig Van Opstal, Jomme Claes, Leen Van Langenhoven, Nicholas Cauwenberghs, Rik Willems, Evangelos Ntalianis, Tatiana Kuznetsova, Veronique Cornelissen, Peter Sinnaeve

**Affiliations:** 1 KU Leuven Department of Cardiovascular Sciences, University of Leuven, Herestraat 49, 3000 Leuven, Belgium; 2 Rehabilitation in Internal Disorders, KU Leuven Department of Rehabilitation Sciences, University of Leuven, Leuven, Belgium; 3 Department of Cardiovascular Diseases, UZ Leuven, Leuven, Belgium; 4 Interuniversity Centre for Biostatistics and Statistical Bioinformatics, KU Leuven and University of Hasselt, Leuven, Belgium

**Keywords:** Cardiopulmonary exercise testing, Ventilatory threshold, Rehabilitation

## Abstract

**Background and Objectives:**

The first ventilatory threshold (VT1) is a key indicator of submaximal aerobic fitness. Its determination typically relies on visual assessment, which is time-consuming and subject to interobserver variability. Automated solutions may offer a more objective, reproducible and time-efficient alternative. This study evaluated the accuracy of a revised V-slope method.

**Methods:**

This retrospective study was conducted at University Hospitals Leuven, Belgium, using cardiopulmonary exercise test (CPET) data of 271 healthy adults collected between 2010 and 2020. Automated VT1 detection using a revised V-slope algorithm was compared with manual determination via the visual V-slope method in 262 participants with a determinable VT1. Agreement was evaluated using Bland–Altman analysis, intraclass correlation coefficients (ICC), Deming regression and equivalence testing (TOST). Interobserver agreement was evaluated using ICC.

**Results:**

Median oxygen uptake at VT1 was higher with automated compared to manual assessment (1151 vs. 1122 mL/min; *p* < 0.001). Mean difference was 29 mL/min (95% CI [19, 40]. Relative mean bias was 2.2% ± 7.1% (LoA [− 11.7 to 16.0%], with excellent agreement (ICC 0.97 [95% CI 0.96–0.98] and a Deming regression slope of 0.95 [95% CI 0.92–0.99] and intercept of 26.1 mL/min [95% CI −11.9 to 64.7]. TOST with equivalence bounds [− 100, 100] was significant (*p *< 0.001; 90% CI [20, 38]).

**Conclusion:**

The revised V-slope algorithm demonstrated strong group-level agreement with visual assessment for VT1 determination, supporting the reliability and practical applicability for large-scale research applications in healthy adults. However, given the observed individual-level variability, caution is warranted when applying automated VT1 values to guide exercise prescription for individual patients without expert review.

**Supplementary Information:**

The online version contains supplementary material available at https://doi.org/10.1186/s40798-026-01124-8.

## Key Points

- Determining the first ventilatory threshold (VT1), marking the transition from moderate- to heavy-intensity exercise, and valued for assessing fitness, prognosis, and exercise prescription, has traditionally relied on visual interpretation by trained experts, a process that is time-consuming and subjective. This study validated a computer algorithm that detects VT1 automatically from exercise test data.
- The automated algorithm showed strong group-level agreement with expert visual assessment in 262 healthy adults, with agreement metrics comparable to those seen between two experienced human raters. However, individual-level differences remained substantial in a subset of participants. The algorithm, therefore, has the potential to reduce analyst workload, improve standardization, and enable large-scale exercise testing analyses, though further validation in patients with heart, lung, or metabolic diseases is needed before routine clinical use.

**Supplementary Information:**

The online version contains supplementary material available at https://doi.org/10.1186/s40798-026-01124-8.

## Introduction

The first ventilatory threshold (VT1) marks the transition from moderate- to heavy-intensity exercise and is widely regarded as a non-invasive surrogate of the lactate or ‘anaerobic’ threshold, reflecting a shift in the balance between lactate production and clearance [1]. Physiologically, VT1 corresponds to the exercise intensity at which carbon dioxide output and, consequently, ventilation increase disproportionately relative to oxygen uptake due to lactate buffering.

Several complementary methods exist to detect VT1. The V-slope method identifies the breakpoint in the VCO_2_–VO_2_ relationship reflecting a disproportionate increase in VCO_2_ relative to VO_2_ [1–5]. Some authors also refer to it as the gas exchange threshold (GET) [1, 4, 6]. This breakpoint can be located either through computerized regression analysis, as in the original V-slope method [3], or visually, either by direct inspection of the curve, or using the modified V-slope method, in which VT1 is identified as the point at which the slope increases from less than 1 to greater than 1 [6]. Other established methods include the ventilatory equivalents method, defined as the first systematic increase in the ventilatory equivalent for oxygen without a simultaneous increase in the ventilatory equivalent for carbon dioxide [2, 4, 5]; the end-tidal pressure method, defined as the first systematic increase in end-tidal oxygen pressure, while end-tidal carbon dioxide pressure remains stable or continues to increase [2, 4]; and the excess carbon dioxide method, in which excess carbon dioxide production increases from a steady-state response to an excess production of CO_2_ during incremental exercise [5].

Despite methodological differences, VT1 is valued as a non-invasive, effort-independent indicator of aerobic fitness with high prognostic value [1, 4, 5]. In addition, exercise prescriptions guided by VT1 have been shown to yield greater improvements in peak VO_2_ and metabolic health than prescriptions based on fixed percentages of peak exercise capacity [2]. Consequently, accurate determination of VT1 is essential both for assessing fitness and prognosis and for guiding individualized exercise prescription.

Traditionally, VT1 is assessed manually during a progressive cardiopulmonary exercise test (CPET) using a combination of the above-mentioned methods, which is generally preferred over reliance on a single criterion [5]. However, when only one method is used for the ease of implementation, the V-slope method appears the most favourable, as it directly captures the rise in CO_2_ production relative to O_2_ uptake arising from bicarbonate buffering, rather than relying on downstream ventilatory changes that may be attenuated by obesity, airflow obstruction, or chemoreceptor insensitivity [3]. When validated against the lactate threshold, the V-slope method also demonstrates the highest acceptance rate of any single measurement method [5]. However, visual assessment relies on expert interpretation and is inherently susceptible to bias. Even among experienced physicians, disagreement in VT1 occurs in approximately 39% of cases, resulting in a variability of 11% in healthy individuals, and up to 18% among less experienced assessors [7]. To address these limitations, automated approaches based on gas exchange kinetics have been proposed, aiming to provide a more objective, reproducible, and time-efficient VT1 determination [3, 7, 8].

Recently, a revised V-slope method building on the original approach of Beaver et al., was proposed and the algorithm was made publicly available [3, 9]. The approach of Beaver et al. identifies the breakpoint in the VCO_2_–VO_2_ relationship by maximizing the deviation of a two-segment linear model from a single linear fit. Rather than iteratively defining a tentative breakpoint, the revised approach locates VT1 at the point of maximum perpendicular distance (PD_max_) between a global linear regression and a quadratic fit to the VCO_2_–VO_2_ curve, thereby mathematically optimizing the same objective function in a fully data-driven manner [9]. Its agreement with manual VT1 determination, however, has not yet been formally assessed. Therefore, the present study aims to validate this automated VT1 detection method against manually determined VT1 values in a large cohort of adults, evaluating group-level agreement, systematic bias and within-subject variability to assess the reproducibility and suitability of implementing automated VT1 detection in CPET workflows. A relatively healthy population was chosen to allow initial evaluation in a population with well-defined physiological response patterns, before extending analyses to clinical populations with more complex ventilatory responses.

## Materials and Methods

### Study Participants

We analysed CPET data from the iCOMPEER study (“Integrative computer modelling for personalized profiling of CRF and prediction of response to ambulatory cardiac rehabilitation”), which includes retrospective data from 3466 individuals who underwent a CPET on a cycle ergometer at University Hospital Leuven (2010–2020) [10]. CPET referral reasons were broad, including pre-exercise programme evaluation for spine rehabilitation or assessment in the pain clinic (Online Resource, Table S1).

Individuals with a history of cardiovascular, pulmonary, or autoimmune disease, diabetes, malignancy, or bariatric surgery were excluded. Additional exclusion criteria were use of antidepressant, anti-inflammatory, or antipsychotic medications or recreational drugs, age ≥80 years, BMI below 18 kg/m^2^, pregnancy, or unavailability of raw CPET time-series data.

### Cardiopulmonary Exercise Test and VT1 Assessment

#### Data Collection

CPETs were performed on a cycle ergometer (Ergometrics 800S) using a stepwise protocol until volitional exhaustion, based on the clinician's pre-test clinical assessment of the participant's expected cardiorespiratory fitness with the aim of achieving an exercise duration of approximately 8–12 min, within the range considered appropriate for VT1 determination [4]. Following an initial one-minute resting period, the standard protocol began at 20 Watts with increments of 20 W/min, targeting a pedalling cadence of 55–65 revolutions per minute. Three CPET systems were used throughout the study, including two breath-by-breath analysers (two from Oxygen AlphaR, Jaeger: one from Med Graphics Ultima, MGC Diagnostics). VO_2_peak was defined as the highest 30-s average at test termination. Predicted VO_2_max was estimated using the Gläser formula [11]. For comparison of VT1 assessment, VO_2_ was preferred over work rate, as the rate of workload increase does not affect the metabolic threshold itself, but does influence the work rate at which VT1 occurs [12, 13]. In addition, comparison of HR at VT1 was performed, as heart rate (HR) at VT1 is a surrogate marker often used in exercise prescription and rehabilitation programs.

#### Signal Processing

In both methods, values were sampled at 10-s intervals and were automatically pre-processed to remove extreme low values at test onset (VCO_2_ ≤ 80 mL/min) and end (VCO_2_ ≤ 350 mL/min), correct abrupt changes (≥ 3 × SD) and replace them by the previous value plus half the absolute difference. Remaining outliers were identified by comparing each value to the mean ± SD of three preceding and three following measurements, and values exceeding this range were replaced by the average of the preceding and following measurements. Signal processing was performed using a custom Python pipeline, as previously described by Ntalianis et al. [14].

#### VT1 Assessment

In the manual method, VO_2_ at VT1 was identified for all tests by two experienced observers through direct visual inspection of the breakpoint in the VCO_2_–VO_2_ curve. Tests in which no discernible breakpoint could be identified, or in which the curve exhibited abnormal behaviour, were classified as indeterminate. When both observers classified a test as indeterminate, the test was excluded from further analysis. When the two observers' classifications of a test as indeterminate disagreed, the observers jointly re-discussed the test to reach consensus. The average of the two independently determined VO_2_ at VT1 values was retained as the final manual value.

**Fig. 1:**
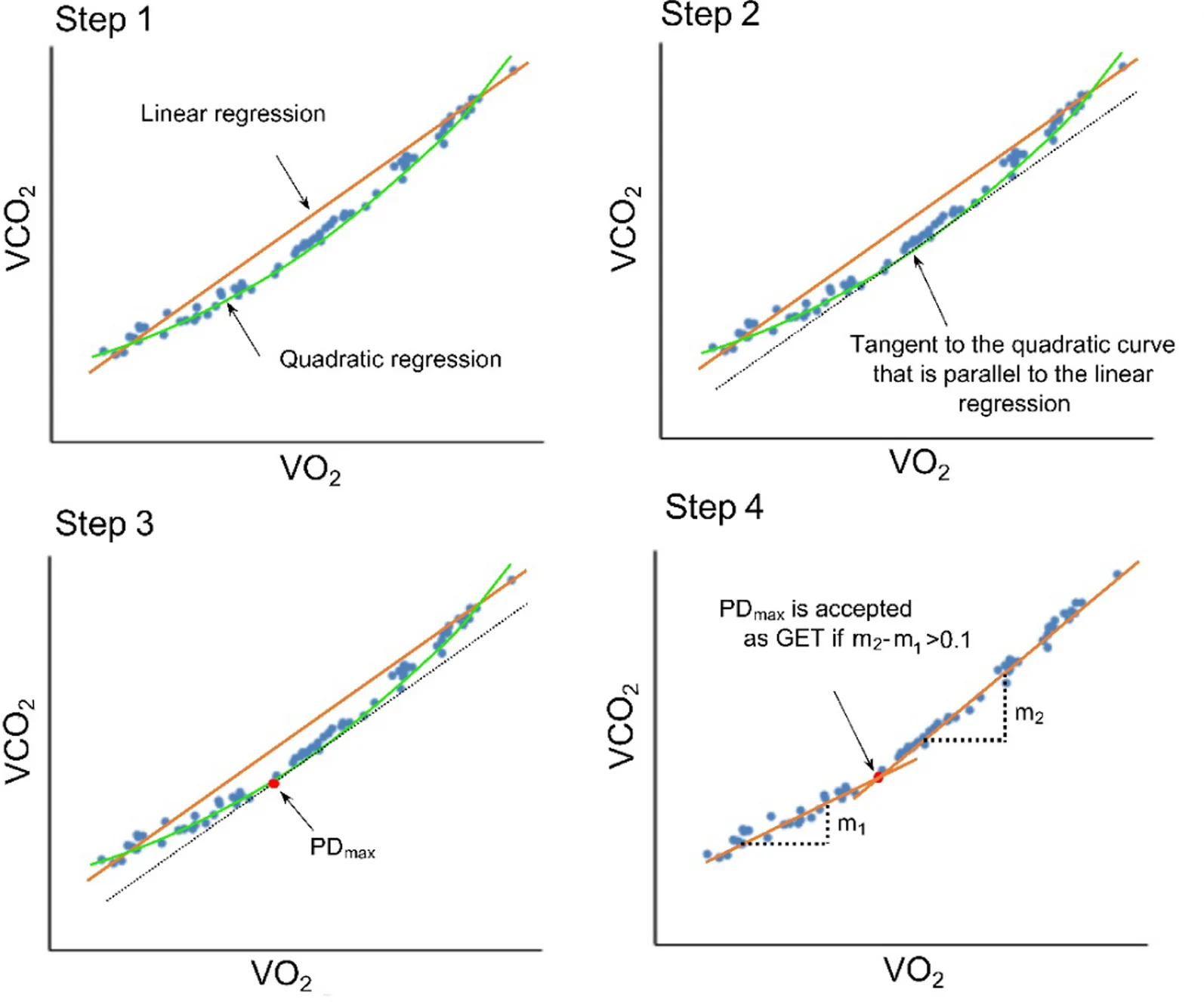
Automated approach to detect VT1. Adapted with permission from Sarthy V. (2020). get_GET: Determination of Ventilatory/Gas Exchange Threshold from a cardiopulmonary exercise test [9]. Step 1: linear (orange) and quadratic (green) regressions are fitted to the exercise VO_2_ and VCO_2_ data. Step 2–3: the tangent to the quadratic curve parallel to the linear fit defines the point of maximum perpendicular distance (PD_max_, red), dividing the data into lower and upper segments. Step 4: PD_max_ is accepted as VT1 when the difference in segment slopes exceeds 0.1

In the automated method, linear and quadratic regressions were fitted to the incremental exercise data only, with resting and recovery data excluded, using the publicly available GET package within a Python 3.12 environment [9]. None of the authors were involved in the development of this software. PD_max_ was determined by identifying the tangent of the quadratic curve parallel to the linear regression, thereby dividing the data into lower and upper segments, each fitted with a separate linear regression (Fig. 1) and was accepted as VT1 if the difference in slope between segments exceeded 0.1, a criterion adopted from the original V-slope method to eliminate spurious breakpoints arising from noise [3]. If this criterion was not met, the algorithm was permitted to consider ±3 neighbouring values.

### Statistics

Continuous variables were summarized as mean ± SD or median [IQR], where appropriate. Normality and skewness were assessed using visual inspection of histograms. Paired measurements were compared using a paired *t*-test, Wilcoxon signed-rank, or sign test, depending on normality and symmetry of the paired differences.

Agreement between methods was assessed using Bland–Altman analysis to evaluate systematic bias and 95% limits of agreement (LoA). LoA were compared to the pre-defined boundary of 100 mL/min, corresponding to the minimal acceptable measurement error for VO_2_ at VT1 [4]. Proportional bias was examined using linear regression of the differences against the mean of both measurements, while heteroscedasticity was assessed by regressing the absolute residuals from the proportional bias model on the mean. In the presence of heteroscedasticity, percentage differences [100 × (manual − automated)/mean] were analysed and plotted against the logarithm of the mean values. Regression analyses were repeated under these transformations. Deming regression was applied to assess proportional and constant bias, taking into account measurement errors in both methods. Slopes and intercepts were reported along with 95% confidence intervals (CI) estimated via 10,000 bootstrap resamples. Formal paired equivalence was tested with the Two One-Sided Tests procedure, using predefined equivalence bounds of ±100 mL/min. Equivalence for HR at VT1 was tested using a pre-specified margin of ±7 bpm, based on the day-to-day standard error of measurement for HR during submaximal exercise testing in healthy adults [15]. Intraclass correlation coefficients (ICC) were calculated using a two-way mixed-effects model (without interactions) for inter-method agreement and using a two-way random-effects model (without interactions) for interobserver reliability. For the latter, both single-measures and average-measures ICCs were reported, given that the final manual VT1 value was defined as the average of the two independent observer determinations. The latter was calculated only for tests without an indeterminate-related disagreement between observers (i.e., prior to consensus discussion). Subgroup analyses were performed according to sex.

Statistical analyses were performed using a Python 3.12 environment with Python libraries for data processing (SciPy, Numpy) and Stata (version 19.5, StataCorp LLC, Texas, US). ICC were calculated using AgreeStat360 (Gwet, K. L.) [16]. A two-sided *p*-value < 0.05 was considered statistically significant. Plots were generated using Matplotlib (within the same Python 3.12 environment) and finalized with Inkscape (version 1.4.3).

## Results

### Cohort Description

Within the iCOMPEER cohort, 813 subjects were eligible, of whom 271 had available raw CPET data. After exclusion of nine subjects (5 with a slope difference < 0.1, 7 with an indeterminate manual assessment, of whom 3 participants met both exclusion criteria), the final cohort included 262 subjects. The mean age was 46 ±13 years and 61% were female. Subjects were predominantly overweight (BMI 26 kg/m^2^ [IQR 23, 30] and demonstrated a good exercise capacity (% predicted VO_2_max 93 ± 18%) (Table 1).

**Table 1:** Descriptives of the cohort (*n* = 262)

Variable				
Age, years	46 ± 13			
Female, n(%)	161 (61)			
Weight, kg	77 [66, 89]			
Height, cm	171 ± 9			
BMI, kg/m^2^	26 [23, 30]			
% predicted VO_2_max, %	93 ± 18			
Resting heart rate, bpm	75 [67, 85]			
Peak heart rate, bpm	164 [150, 176]			
VO_2_peak, mL/min	1807 [1408, 2209]			
VO_2_peak, mL/kg/min	23.8 [19.2, 28.9]			
Peak RER	1.15 [1.12, 1.20]			
Peak load, W	160 [140, 200]			
CPET duration, seconds	505 ± 156			
Type of exercise protocol				
10 + 10 W/min, n(%)	25 (10)			
20 + 10 W/min, n(%)	10 (4)			
20 + 15 W/min, n(%)	3 (1)			
20 + 20 W/min, n(%)	224 (86)			

	Automated	Manual	Automated-manual	*p*-value, [95% CI]
VO_2_ at VT1, mL/kg/min	15.2 [12.6, 17.7]	14.8 [12.4, 17.6]	0.4 ± 1.1	*p* < 0.001 [0.2, 0.5]
VO_2_ at VT1, mL/min	1151 [953, 1399]	1122 [934, 1351]	29 ± 87	*p* < 0.001 [19, 40]
VO_2_ at VT1/VO_2_peak, %	62 ± 4	63 ± 7	1 ± 4	*p* < 0.001 [1, 2]
VO_2_ at VT1/predicted VO_2_max, %	58 ± 10	58 ± 10	1 ± 4	*p* < 0.001 [1, 2]
Heart rate at VT1, bpm^a^	114 ± 17	114 ± 17	4 ± 8	*p* < 0.001 [3, 5]
Load at VT1, W	100 [80, 140]	80 [60, 100]	5 ± 14	*p* < 0.001 [3, 7]

Data are presented as mean ± SD, median [IQR], or *n*(%)

BMI, body mass index; bpm, beats per minute; CI, confidence interval; CPET, cardiopulmonary exercise test; RER, respiratory exchange ratio; VO_2_, oxygen uptake; VT1, first ventilatory threshold; W, Watts

^a^1 record was excluded due to erroneous heart rate measurements

Oxygen uptake at VT1 was higher with automated compared to manual assessment (29 ± 87 mL/min (95% CI [19, 40])). A similar pattern was seen for VO_2_ at VT1 relative to VO_2_peak (1 ± 4%) and for HR at VT1 (4 ± 8 bpm). Descriptive data stratified by sex are provided in Online Resource Tables S2 and S3. Descriptive values for the underlying mathematical parameters and their comparison are provided in the Online Resources, Table S5.

### Intraclass Correlations

**Fig. 2:**
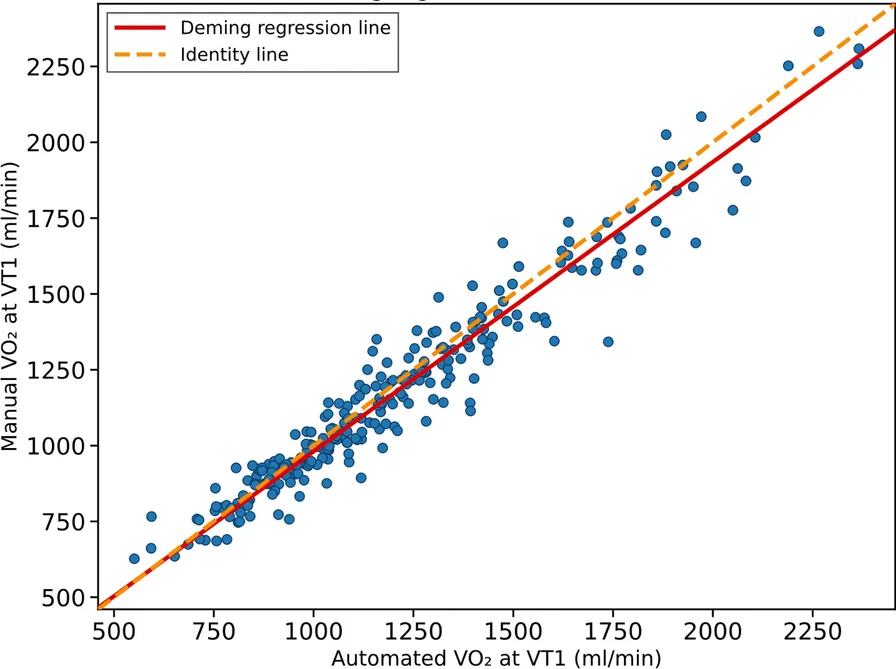
Scatter plot with Deming Regression line (*n* = 262)**.** Scatter plot illustrating the relationship between automated and manual VO_2_ at VT1. The red solid line represents the Deming regression line (slope 0.95, 95% CI 0.92–0.99; intercept 26.1 mL/min, 95% CI −11.9 to 64.7); the orange dashed line represents the identity line. Each dot represents one participant. Abbreviations: VO_2_, oxygen uptake: VT1; first ventilatory threshold

Inter-method agreement was excellent, with an ICC of 0.97 [95% CI 0.96–0.98; *p* < 0.001] overall, and similarly high within sex-stratified subgroups (men: 0.96 [95% CI 0.93–0.97]; women: 0.94 [95% CI 0.92–0.96]). Deming regression showed a slope of 0.95 [95% CI 0.92–0.99] and an intercept of 26.1 mL/min [95% CI −11.9 to 64.7], indicating proportional bias, whereby automated values were higher than manual values at higher VO_2_ levels, without evidence of constant bias (Fig. 2). Scatter plots stratified by sex are provided in Online Resource, Figures S1 and S2.

### Bias and Limits of Agreement

**Fig. 3:**
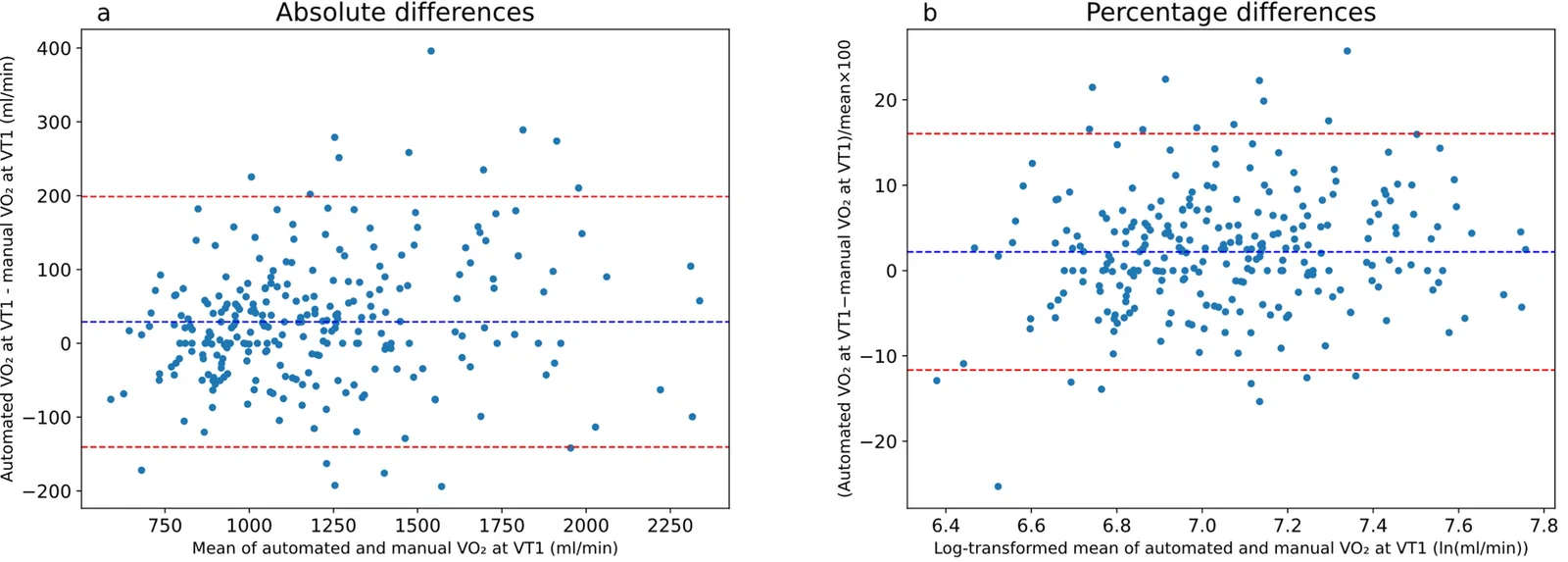
Bland–Altman plots comparing automated and manual determination of VO_2_ at VT1 (*n* = 262). **a** Absolute differences between methods. The mean bias was 29 mL/min (blue dashed line), with 95% LoA ranging from −141 to +199 mL/min (red dashed lines). **b** Percentage differences between methods. The mean bias was 2.2% (blue dashed line), with 95% LoA ranging from −11.7 to +16.0% (red dashed lines). Abbreviations: VO_2_, oxygen uptake; VT1, first ventilatory threshold

Bland–Altman analysis of absolute differences (Fig. 3a) revealed a mean bias of 29 mL/min, with 95% LoA ranging from −140 to +199 mL/min, exceeding the pre-specified boundary of 100 mL/min. Overall, 79% of participants had a difference within the margin, with men 66% within margin and women 86% within margin (Online Resources, Table S4). Proportional bias was detected (slope = 0.05, *p* = 0.002) and heteroscedasticity was present (slope of absolute residuals = 0.06, *p* < 0.001), warranting analysis of percentage differences (Fig. 3b). After this transformation, no heteroscedasticy was present (slope − 0.61, *p* = 0.641), however, proportional bias remained (slope = 3.63, *p* = 0.023). The mean percentage difference was 2.2% ± 7.1%, with 95% LoA ranging from − 11.7 to +16.0%. Bland–Altman plots stratified by sex are provided in Online Resource, Figures S3 and S4.

### Equivalence

Equivalence testing with a pre-specified margin of 100 mL/min was significant, *p* < 0.001; the 90% CI [20, 38] mL/min fell entirely within the equivalence region. Equivalence was observed in both men (*p* < 0.001) and women (*p* < 0.001) (Online Resources, Table S4).

### Interobserver Variability

Interobserver reliability for the manual method was good, with a single-measure ICC of 0.93 [95% CI 0.90–0.95] (*n* = 256). The average-measures ICC was excellent at 0.96 [95% CI 0.95–0.97]. Relative mean bias between observer 2 and observer 1 was − 3.0%, with 95% LoA of − 16.2% to 22.1%, proportional bias was present (*p* = 0.003); however, no heteroscedasticity (*p* = 0.280) was detected.

### Heart Rate at VT1

Among 261 subjects (1 excluded for erroneous HR measurements), agreement between automated and manual HR at VT1 was good (ICC, 0.91; 95% CI 0.89–0.93). Deming regression yielded a slope of 0.94 (95% CI 0.90–0.98) and an intercept of 3.6 bpm (95% CI −1.6 to 8.4). On Bland–Altman analysis, automated HR was on average 4 bpm higher than manual HR (95% limits of agreement, −10 to 18 bpm); there was proportional bias (slope = 0.07, *p* 0.011) and heteroscedasticity (*p* < 0.001). Analysis of percentage differences showed a mean bias of 3.1% (LoA, −8.6 to 14.8%), with proportional bias (slope = 4.91, *p* = 0.047); however, no heteroscedasticity was present (*p* = 0.185). Equivalence testing with a margin of ±7 bpm was significant (90% CI [3, 5] bpm, *p* < 0.001); 75% of participants had a difference within this margin (78% of men and 73% of women). Full results are shown in the Online Resource (Table S4, Figures S5–S8).

## Discussion

The first ventilatory threshold (VT1) is a key determinant of submaximal aerobic fitness and plays a central role in exercise prescription, with the added advantage of being largely independent of subject motivation. Visual assessment, however, is time-consuming, inherently subjective, and prone to interobserver variability, although this can be partly mitigated by following a standardized assessment protocol [4]. These limitations, combined with the demands of large-scale data analysis, underscore the need for reliable automated detection algorithms. The present study constitutes the first formal validation of the revised V-slope algorithm proposed by Sarthy et al. against manually determined VT1 values in a large cohort of relatively healthy adults [9].

The automated algorithm demonstrated strong agreement with visual assessment at the group-level, as evidenced by equivalence testing and a high ICC. However, Bland–Altman analysis revealed substantion individual-level variability. Deming regression indicated a small proportional bias (slope 0.95), whereby automated VT1 estimates were progressively higher relative to manual values at higher VO_2_ levels, without evidence of significant constant bias. Individuals with higher exercise capacity typically progress through higher workload steps, resulting in larger absolute increments in VO_2_ between consecutive stages. Hypothetically, if the algorithm has a tendency to overestimate VT1 across individuals, this could translate into larger absolute differences in mL/min at higher VO_2_ values. Heteroscedasticity in the Bland–Altman analysis of absolute differences indicated that measurement error scales with the magnitude of VO_2_, a pattern commonly observed in physiological measurements. This justified the use of percentage differences as the agreement metric; although heteroscedasticity was no longer present after this transformation, a modest proportional bias persisted (slope = 3.63, *p* = 0.023).

The algorithm failed to identify a valid VT1 in approximately 2% of cases. Notably, the majority of these were also indeterminate on manual assessment, suggesting that algorithmic failures may reflect inherently ambiguous VCO_2_–VO_2_ relationships rather than a specific limitation of the automated method.

These results compare favourably with previously reported validations of automated VT1 detection against visual assessment. A software-based tool applied in over 500 asymptomatic healthy volunteers reported a mean difference of 22% ± 26%, with LoA exceeding ±800 mL/min, substantially wider than those observed in the present study [7]. A convolutional neural network algorithm reported an average difference of 11% ± 18%, likewise exceeding the mean percentage difference of 2.2% ± 7.1% observed here [8]. A fine-tuned version of this convolutional neural network, tested in 50 healthy participants, reported a mean bias of 45 mL/min with 95% LoA of −313 to 403 mL/min and an ICC of 0.91, comparable to the results observed in the present study [17]. However, direct comparison across studies is further complicated by differences in the manual reference standard, CPET protocols and data pre-processing procedures [18]. Whereas the present study used the visual V-slope method to ensure methodological alignment with the automated algorithm, both targeting the breakpoint in the VCO_2_–VO_2_ curve, other studies relied on a consensus of multiple detection methods [7, 8, 17].

Notably, the level of agreement between the automated method and manual assessment in the present study (2.2% ± 7.1%) closely approached the inter-rater variability reported between experienced cardiologists in a cohort of 522 tests (0% ± 11%) [7]. In this study, the inter-method agreement (ICC 0.97 [95% CI 0.96 to 0.98]) was at least comparable to, and numerically higher than the inter-rater agreement (ICC 0.93 [95% CI 0.90–0.95]; 3.0 ± 10%).

Overall, agreement between methods was higher in women than in men, though this pattern was not observed for the ICC specifically, likely reflecting the greater between-subject variance in men. One possible explanation is the more frequent use of protocols with smaller work-rate increments in women, which may yield finer physiological resolution around the threshold.

As with VO_2_, agreement for HR at VT1 was strong overall. Although the ICC was somewhat lower than for VO_2_ (0.91 vs. 0.97), variability was in fact lower, with a more narrow percentage LoA for HR (− 8.6 to +14.8%) than for VO_2_ (− 11.7 to +16.0%). Of note, the sex-related pattern for HR agreement was the reverse of that seen for VO_2_, with agreement being marginally better in men than in women. Given the generally smaller stroke volume in women, a proportionally greater chronotropic response may be required to achieve a given VO_2_, resulting in a steeper local HR–VO_2_ slope that could amplify small discrepancies in the identified VT1 point into larger HR differences [19].

### Outliers and Sources of Variability

Despite the overall strong agreement, inter-method variability remained, as reflected by the relatively wide limits of agreement and the finding that 21% of participants fell outside the ±100 mL/min margin. This degree of variability is not unique to automated approaches as noted above and likely reflects the inherent ambiguity of threshold identification in certain curve morphologies. Visual examination of outlier curves identified several potential mechanisms underlying this residual variability (Online Resource, Figures S11–S12).

When the VCO_2_–VO_2_ relationship follows a near-linear trajectory throughout the test, identification of a clear breakpoint is inherently difficult for both approaches. Similarly, large increments in VO_2_ near the expected threshold reduce data resolution in the critical region, compromising accuracy irrespective of method. A third source of discordance arises in curves lacking a sharp inflection: the manual method tends to identify the first systematic deviation from linearity, whereas the automated algorithm, anchored to the point of maximum curvature, may select a later point when the transition is gradual. Importantly, visual identification of this first deviation is itself inherently subjective, and re-evaluation of some outliers indicated that the threshold identified by the algorithm was in fact acceptable. Artefactual data points represent an additional source of error, as these can distort regression fitting and bias automated detection, while an experienced evaluator may implicitly discount such values during visual inspection. Finally, an early VT1 leaves a disproportionately large number of data points at higher VO_2_ values, which may shift the mathematically derived breakpoint rightward. An approach combining multiple VT1 detection methods might mitigate some of these limitations. However, even when the manual reference standard incorporates multiple methods, including the V-slope, ventilatory equivalents and end-tidal oxygen pressure, a comparable degree of bias between automated and manual detection seems to persist [17].

### Strengths and Limitations

Several methodological strengths merit consideration. The validation was performed in a well-characterised cohort of relatively healthy adults spanning a broad age range with a balanced sex distribution, enhancing the generalisability of the findings. Both manual and automated assessments were applied to identically pre-processed data, minimising confounding from signal noise. Manual determination was performed independently by two experienced observers for all tests, strengthening the reliability of the reference standard beyond a single-rater assessment. Finally, the use of multiple complementary statistical approaches provides a comprehensive characterisation of method agreement that extends beyond simple correlation statistics.

However, some limitations warrant acknowledgment. Because the algorithm relies on linear regression across the entire exercise data, its result may depend on the degree of maximal effort achieved. Importantly, the study was conducted exclusively in a relatively healthy adult population. Generalisability to clinical groups in whom VT1 determination is inherently more challenging, including patients with heart failure, pulmonary disease, or metabolic disorders, remains to be established and represents an important avenue for future research. Finally, we did not directly compare the revised V-slope algorithm with alternative automated methods for VT1 determination within the same cohort. Such head-to-head comparisons would be valuable to better characterise the relative performance and applicability of this revised method in comparison to other automated approaches to VT1 determination.

### Clinical Implications

The present findings suggest that automated detection may serve as a viable alternative to visual assessment in relatively healthy adults for standardised large-scale CPET analysis to reduce analyst workload, particularly where expert availability is limited or high throughput is required. However, the wide individual-level limits of agreement indicate that, for individual patients, automated VT1 estimates should ideally be reviewed by an experienced assessor before being used to guide exercise prescription, particularly given the clinical importance of an accurate individual threshold.

## Conclusions

The revised V-slope algorithm demonstrated strong group-level agreement with visual assessment for determining VT1 in relatively healthy adults, with inter-method variability comparable to that observed between experienced human raters, suggesting that the uncertainty introduced by the algorithm may be comparable to that observed between experienced human raters. These findings support the use of automated VT1 detection as a standardized, reproducible, and time-efficient tool for large-scale research applications, where some degree of individual-level disagreement can be tolerated. However, the observed LoA indicate that individual estimates may differ meaningfully from expert assessment in a non-negligible proportion of cases. Caution is, therefore, warranted before using automated VT1 values, in isolation, to guide individualized exercise prescription for a given patient. Validation in clinical populations, together with strategies to flag or review possible discordant individual cases, represents an essential next step before broader implementation in routine clinical care.

## Supplementary Information

Supplementary Material 1: Table S1: Reasons for CPET referral. Table S2: Descriptives of the male cohort.Table S3: Descriptives of the female cohort.Table S4: Equivalence testing. Table S5: Mathematical parameters of the algorithm. Figure S1: Deming regression analysis for VO_2_ at VT1in the male cohort.Figure S2: Deming regression analysis for VO_2_ at VT1 in the female cohort. Figure S3: Bland–Altman Plot for VO_2_ at VT1 in the male cohort. Figure S4: Bland–Altman Plot for VO_2_ at VT1 in the female cohort. Figure S5: Bland–Altman Plot for HR at VT1 in the overall cohort. Figure S6: Bland–Altman Plot for HR at VT1 in the male cohort. Figure S7: Bland–Altman Plot for HR at VT1 in the female cohort. Figure S8: Deming regression for HR at VT1 in overall cohort. Figure S9: Deming regression for HR at VT1 in male cohort. Figure S10: Deming regression for HR at VT1 in the female cohort. Figure S11: VCO_2_–VO_2_ curves of outlier cases according to Bland Altman analysis of absolute differences. Figure S12: VCO_2_–VO_2_ curves of outlier cases according to Bland Altman analysis of percentage differences.

## Data Availability

The data underlying this article cannot be shared publicly due to ethical reasons. The data will be shared upon reasonable request to the corresponding author. Both the data cleaning code and the algorithm used for automated detection of the first ventilatory threshold are openly available via GitHub [9, 14].

## Abbreviations

BMI: Body mass index
bpm: Beats per minute
CI: Confidence interval
CPET: Cardiopulmonary exercise test
GET: Gas exchange threshold
HR: Heart rate
ICC: Intraclass correlation coefficient
IQR: Interquartile range
LoA: Limits of agreement
PDmax: Point of maximum perpendicular distance
RER: Respiratory exchange ratio
SD: Standard deviation
VCO_2_: Carbon dioxide output
VO_2_: Oxygen uptake
VT1: First ventilatory threshold
W: Watts
