# Supplementary material for: Validation of a Revised V-Slope Algorithm for Automated First Ventilatory Threshold Detection in Healthy Adults
Source: Sports Med Open. 2026 Oct 11;12:153. doi: 10.1186/s40798-026-01124-8 (PMC13654909; doi:10.1186/s40798-026-01124-8)
Supplement: Supplementary file 1 [file 40798_2026_1124_MOESM1_ESM.pdf]

SUPPLEMENTARY INFORMATION

Validation of a revised V-slope algorithm for automated first ventilatory threshold detection in healthy adults

Margot Vermeiren MD[a,c], Kaatje Goetschalckx MD[a,c], Herwig Van Opstal Msc[b], Jomme Claes PhD[b], Leen Van Langenhoven[d], Nicholas Cauwenberghs PhD[a], Rik Willems MD PhD[a,c], Evangelos Ntalianis PhD[a], Tatiana Kuznetsova MD PhD[a], Veronique Cornelissen PhD[b], Peter Sinnaeve MD PhD[a,c]

[a]KU Leuven Department of Cardiovascular Sciences, University of Leuven, Leuven, Belgium; [b]Rehabilitation in Internal Disorders, KU Leuven Department of Rehabilitation Sciences, University of Leuven, Leuven, Belgium; [c]Department of Cardiovascular Diseases, UZ Leuven, Leuven, Belgium, [d]Interuniversity Centre for Biostatistics and Statistical Bioinformatics, KU Leuven and University of Hasselt, Leuven, Belgium

Sports Medicine - Open

Corresponding author: Margot Vermeiren, margot.vermeiren@kuleuven.be, Herestraat 49, 3000 Leuven, +3216 34 80 30 https://orcid.org/0009-0008-6886-4215

Supplementary Tables

Table S1: Reasons for CPET referral (n = 262)

CPET indication	
Spine rehabilitation	170 (65)
Pain clinic	29 (11)
Obesity	28 (11)
Dyspnea	17 (6)
Hypertension	7 (4)
Deconditioning	7 (4)
Chronic Fatigue Syndrome	4 (2)

Table S1 Data are presented as n(%). CPET, cardiopulmonary exercise test.

Table S2: Descriptives of the male cohort (n = 101)

Variable				
Age, years	49 [37, 56]			
Weight, kg	82 [74, 90]			
Height, cm	179 ± 8			
BMI, kg/m^2	26 [23, 28]			
% predicted VO_2max, %	93 ± 19			
Resting heart rate, bpm	71 [63, 78]			
Peak heart rate, bpm	160 ± 22			
VO_2peak, ml/min	2424 ± 656			
VO_2peak, ml/kg/min	29.4 ± 7.9			
Peak RER	1.17 ± 0.07			
Peak load, W	209 ± 56			
CPET duration, seconds	594 ± 163			
Type of exercise protocol				
10 +10 W/min	4 (4)			
20 + 20 W/min	97 (96)			
	**Automated**	**Manual**	**Automated - Manual**	**p-value, [95% CI]**
VO_2 at VT1, ml/kg/min	17.5 [15.2, 20.7]	16.8 [15.1, 19.7]	0.5 [-0.3, 1.3]	p < 0.001 [0.2, 0.8]
VO_2 at VT1, ml/min	1493 ±352	1444 ±343	49 ± 104	p < 0.001 [28, 69]
VO_2 at VT1/VO_2max, %	62 ± 4	61 ± 7	2 ± 4	p < 0.001

				[1, 3]
VO_2 at VT1/predicted VO_2max, %	58 ± 10	56 ± 10	2± 4	$p < 0.001$ [1, 3]
Heart rate at VT1, bpm	114 ± 17	111 ± 16	2 [0, 7]	$p < 0.001$ [1, 5]
Load at VT1, W	109 ± 32	102 ± 28	8 ± 16	$p < 0.001$ [5, 11]

Table S2 Data are presented as mean ± SD, median [IQR], or n(%). Abbreviations: BMI, body mass index; bpm, beats per minute; CI, confidence interval; CPET, cardiopulmonary exercise test; RER, respiratory exchange ratio; VO_2, oxygen uptake; VT1 = first ventilatory threshold; W = Watts.

Table S3: Descriptives of the female cohort (n = 161)

**Variable**				
Age, years	45 ± 12			
Weight, kg	72 [63, 86]			
Height, cm	167 ± 7			
BMI, kg/m^2	26 [23, 31]			
% predicted VO_2max, %	92 ± 17			
Resting heart rate, bpm	78 [68, 90]			
Peak heart rate, bpm	162 ± 21			
VO_2peak, ml/min	1601 ± 362			
VO_2peak, ml/kg/min	21.3 [17.8, 25.2]			
Peak RER	1.15 ± 0.07			
Peak load, W	148 ± 35			
CPET duration, seconds	430 [360, 520]			
Type of exercise protocol				
10 +10 W/min	21 (13)			
20 + 10 W/min	10 (6)			
20 + 15 W/min	3 (2)			
20 + 20 W/min	127 (79)			
	**Automated**	**Manual**	**Automated - Manual**	**p-value, [95% CI]**
VO_2 at VT1, ml/kg/min	13.8 [11.9, 16.4]	14.0 ± 3.4	0.2 ± 1.0	p = 0.015 [0.0, 0.4]
VO_2 at VT1, ml/min	1035 ± 210	1019 ± 203	17 ± 71	p = 0.004

				[5, 28]
VO_2 at VT1/VO_2max, %	65 ± 5	64 ± 6	1 ± 5	p = 0.017<br>[0, 2]
VO_2 at VT1/predicted VO_2max, %	60 ± 10	59 ± 10	1 ± 5	p = 0.014<br>[0, 1]
Heart rate at VT1, bpm*	119 ± 18	115 ±17	4 ± 9	p < 0.001<br>[3, 5]
Load at VT1, W	75 ± 19	72 ± 18	0 [0, 10]	p = 0.001<br>[0, 0]

Table S3 Data are presented as mean ± SD, median [IQR], or n(%). Abbreviations: BMI, body mass index; bpm, beats per minute; CI, confidence interval; CPET, cardiopulmonary exercise test; RER, respiratory exchange ratio; VO_2, oxygen uptake; VT1, first ventilatory threshold; W, Watts. * 1 record was excluded due to erroneous heart rate measurements.

Table S4: Equivalence testing

Variable	Statistic	Overall	Male cohort	Female cohort
VO_2 at VT1 (±100 mL/min) (Cohen's d 1.155)	TOST p-value	< 0.001	< 0.001	< 0.001
	90% CI of mean difference	20 to 38 ml/min	32 to 66 ml/min	7 to 26 ml/min
	% within margin	79%	66%	86%
	Observed Cohen's d	0.34	0.47	0.23
HR at VT1 (± 7bpm) (Cohen's d 0.896)	TOST p-value	< 0.001	< 0.001	< 0.001
	90% CI of mean difference	3 to 5 bpm	3 to 5 bpm	3 to 5 bpm
	% within margin	75%	78%	73%
	Observed Cohen's d	0.49	0.61	0.45

Table S4 Equivalence between automated and manual VT1 assessment was evaluated using the Two One-Sided Tests (TOST) procedure with equivalence margins of ±100 mL/min for VO_2 at VT1 and ±7 bpm for HR at VT1. The observed Cohen's d is reported as a standardized measure of effect size for the paired differences, calculated directly from our data as the mean difference divided by the standard deviation of the paired differences. Equivalence was demonstrated overall and within both sexes for both VO_2 and HR (all $p < 0.001$). Abbreviations: bpm, beats per minute; CI, confidence interval; HR, heart rate; VO_2, oxygen uptake; VT1, first ventilatory threshold.

Table S5: Mathematical parameters of the algorithm (n = 262)

Variable		p-value
**Segment slopes**		
m1 (pre-threshold slope)	0.93 ± 0.14	
m2 (post-threshold slope)	1.54 [1.49, 1.57]	
m1 vs. m2 (m2-m1)	0.62 [0.56, 0.66]	<0.001
**Reference line slopes**		
m3 (overall lineair regression slope)	1.28 ± 0.12	
m4 (Dmax end-line slope)	1.27 ± 0.14	
m3 vs. m4 (m4 – m3)	-0.01 ± 0.08	0.758

Table S5 Values are presented as mean ± SD or median [IQR]. The post-threshold slope (m2) was significantly steeper than the pre-threshold slope (m1). The slope of the overall linear regression (m3) did not differ significantly from the slope of the line connecting the first and last data points of the curve (m4, corresponding to the classical Dmax reference line (1)).

Supplementary Figures

Figure S1: Deming regression analysis for VO_2 at VT1 in the male cohort (n = 101)

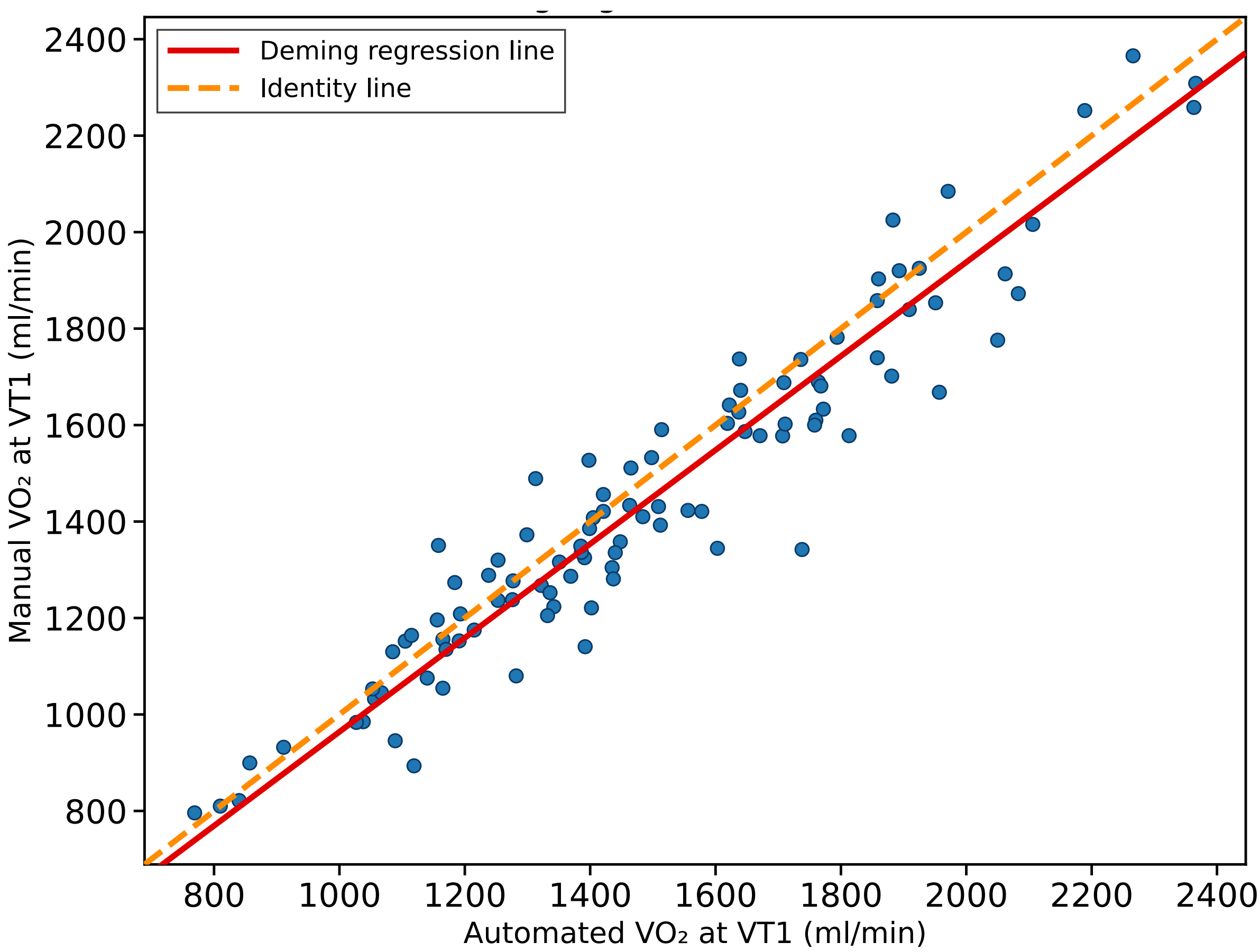

Fig. S1 Scatter plot illustrating the relationship between automated and manual VO_2 at VT1 in the male cohort. The red solid line represents the Deming regression line (slope 0.97, 95% CI 0.92–1.03; intercept -9.6 ml/min, 95% CI -89.9 to); the orange dashed line represents the identity line. Each dot represents one subject. Abbreviations: VO_2, oxygen uptake; VT1, first ventilatory threshold.

Figure S2: Deming regression analysis for VO_2 at VT1 in the female cohort (n = 161)

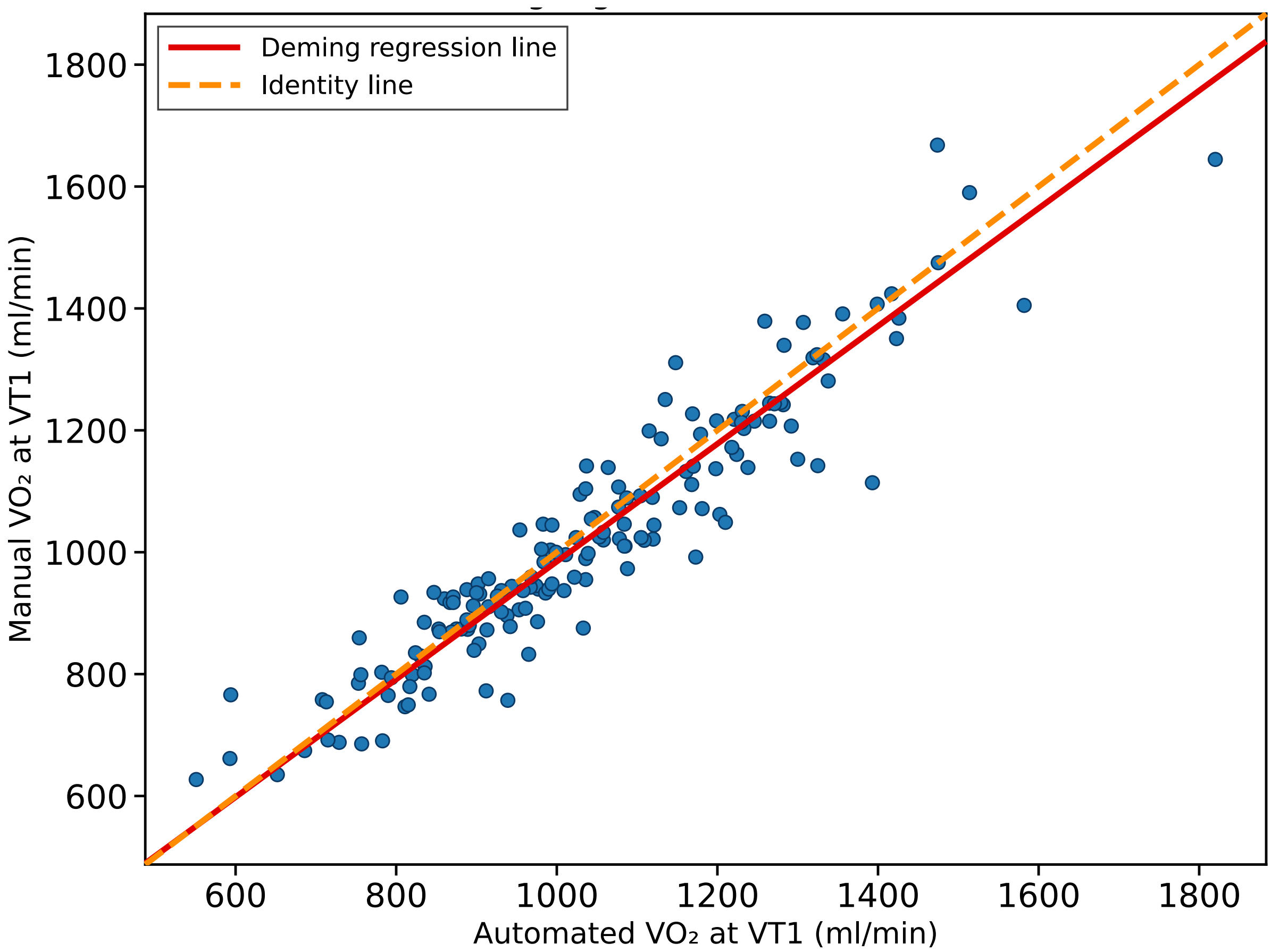

Fig. S2 Scatter plot illustrating the relationship between automated and manual VO_2 at VT1 in the female. The red solid line represents the Deming regression line (slope 0.97, 95% CI 0.90–1.04; intercept 18.5 ml/min, 95% CI -51.2 to 80.8); the orange dashed line represents the identity line. Each dot represents one subject. Abbreviations: VO_2, oxygen uptake; VT1, first ventilatory threshold.

Figure S3: Bland-Altman Plot for VO_2 at VT1 in the male cohort (n = 101)

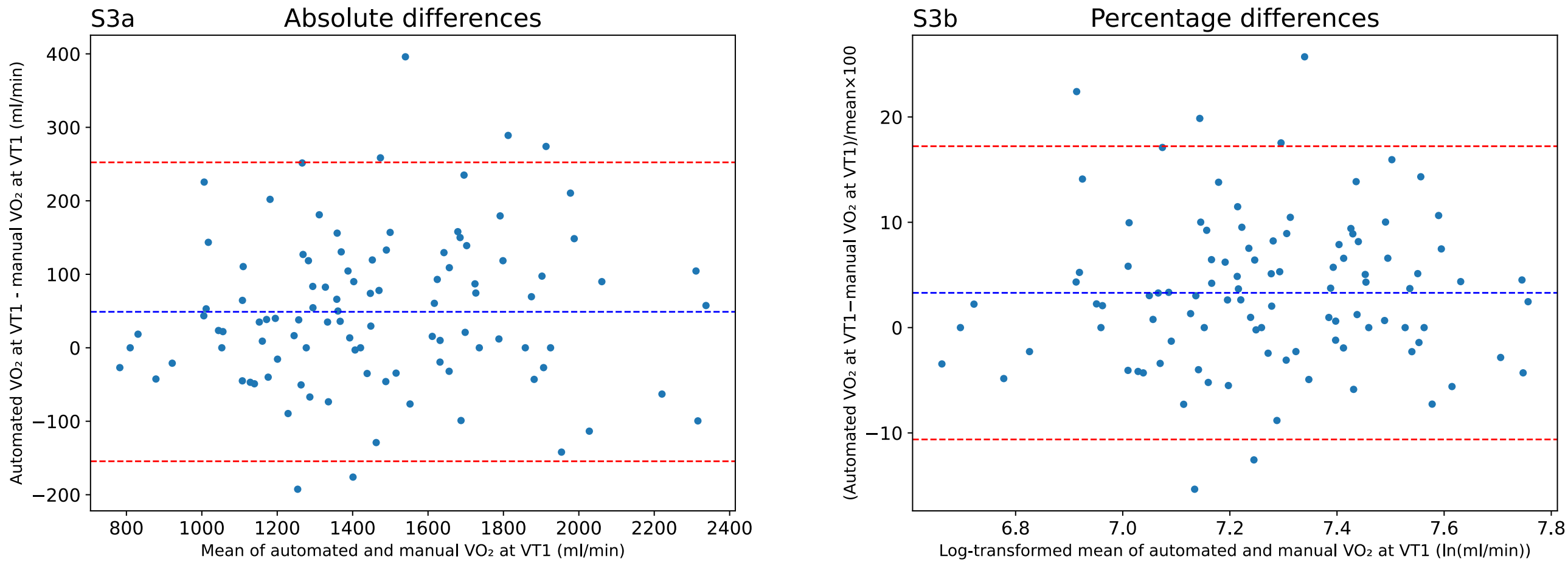

Fig. S3 (a) Absolute differences between methods. The mean bias was 48.9 ml/min (blue dashed line), with 95% LoA ranging from −154.5 to 252.3 ml/min (red dashed lines). No proportional bias was detected (slope = 0.03, $p = 0.369$); however, heteroscedasticity was present (slope of absolute residuals = 0.05, $p = 0.047$). **(b)** Percentage differences between methods. The mean percentage difference was 3.3% ± 7.1% (blue dashed line), with 95% LoA ranging from −10.6 to +17.2% (red dashed lines). Neither proportional bias (slope = 1.18, $p = 0.695$) nor heteroscedasticity (slope of absolute residuals = -0.61, $p = 0.801$) was present. Abbreviations: VO_2, oxygen uptake; VT1, first ventilatory threshold.

Figure S4: Bland-Altman Plot for VO_2 at VT1 in the female cohort (n = 161)

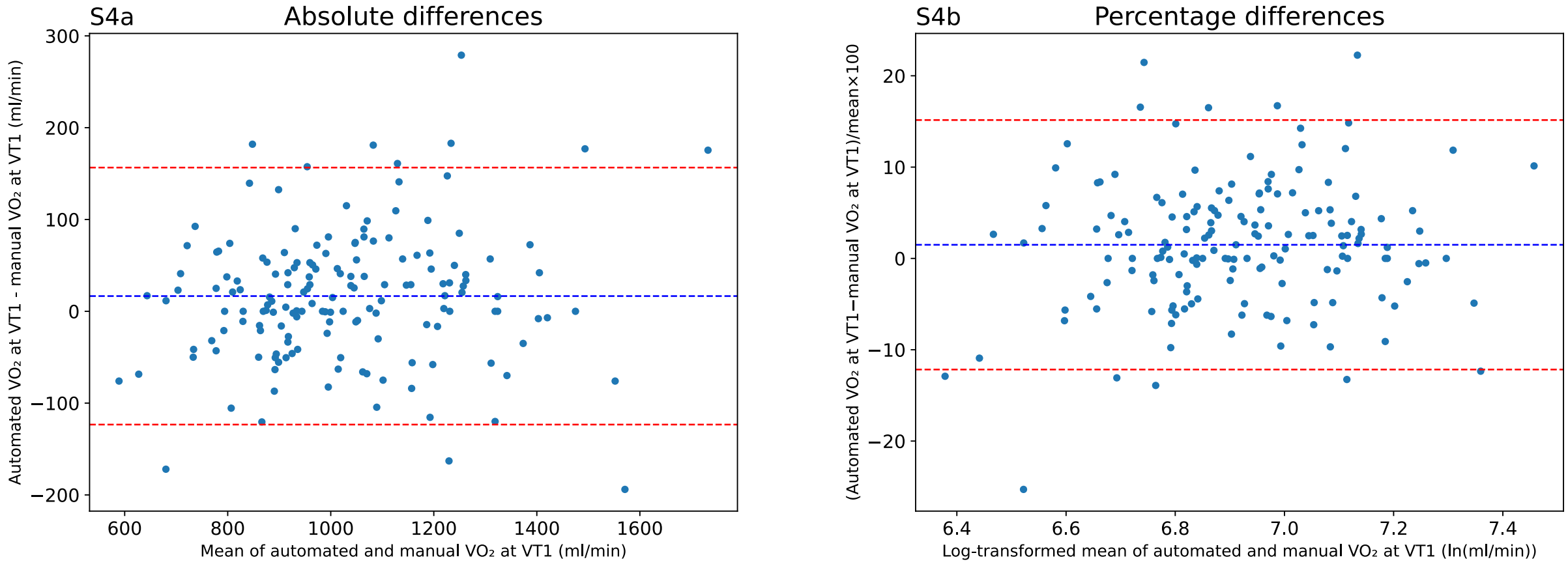

Fig. S4 (a) Absolute differences between methods. The mean bias was 16.6 ml/min (blue dashed line), with 95% LoA ranging from −123.4 to 156.6 ml/min (red dashed lines). No proportional bias was detected (slope = 0.04, p = 0.201); however, heteroscedasticity was present (slope of absolute residuals = 0.06, p = 0.009). **(b)** Percentage differences between methods. The mean percentage difference was 1.5% ± 7.0% (blue dashed line), with 95% LoA ranging from −12.2 to +15.2% (red dashed lines). No proportional bias (slope = 3.96, p = 0.158) nor heteroscedasticity (slope = -2.72, p = 0.235) was present. Abbreviations: VO_2, oxygen uptake; VT1, first ventilatory threshold.

Figure S5: Bland-Altman Plot for HR at VT1 in the overall cohort (n = 261)

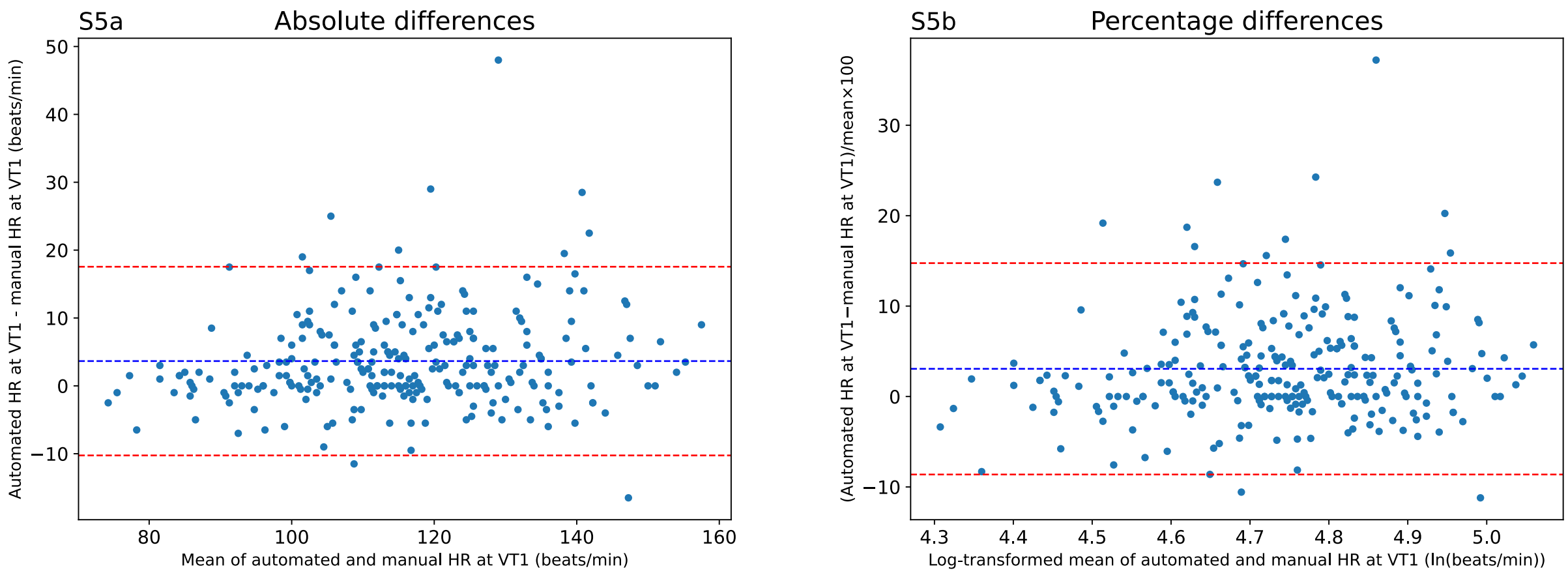

Fig. S5 (a) Absolute differences between methods. The mean bias was 3.6 beats/min (blue dashed line), with 95% LoA ranging from -10.3 to 17.6 (red dashed lines). Both proportional bias (slope = 0.07, p = 0.011) and heteroscedasticity were present (slope of absolute residuals = 0.07, p < 0.001). **(b)** Percentage differences between methods. The mean percentage difference was 3.1% ± 6.0% (blue dashed line), with 95% LoA ranging from −8.6 to +14.8% (red dashed lines). Proportional bias was present (slope = 4.91, p = 0.047);but no heteroscedasticity was detected (slope = 2.80, p = 0.185). Abbreviations: HR, heart rate; VT1, first ventilatory threshold.

Figure S6: Bland-Altman Plot for HR at VT1 in the male cohort (n = 101)

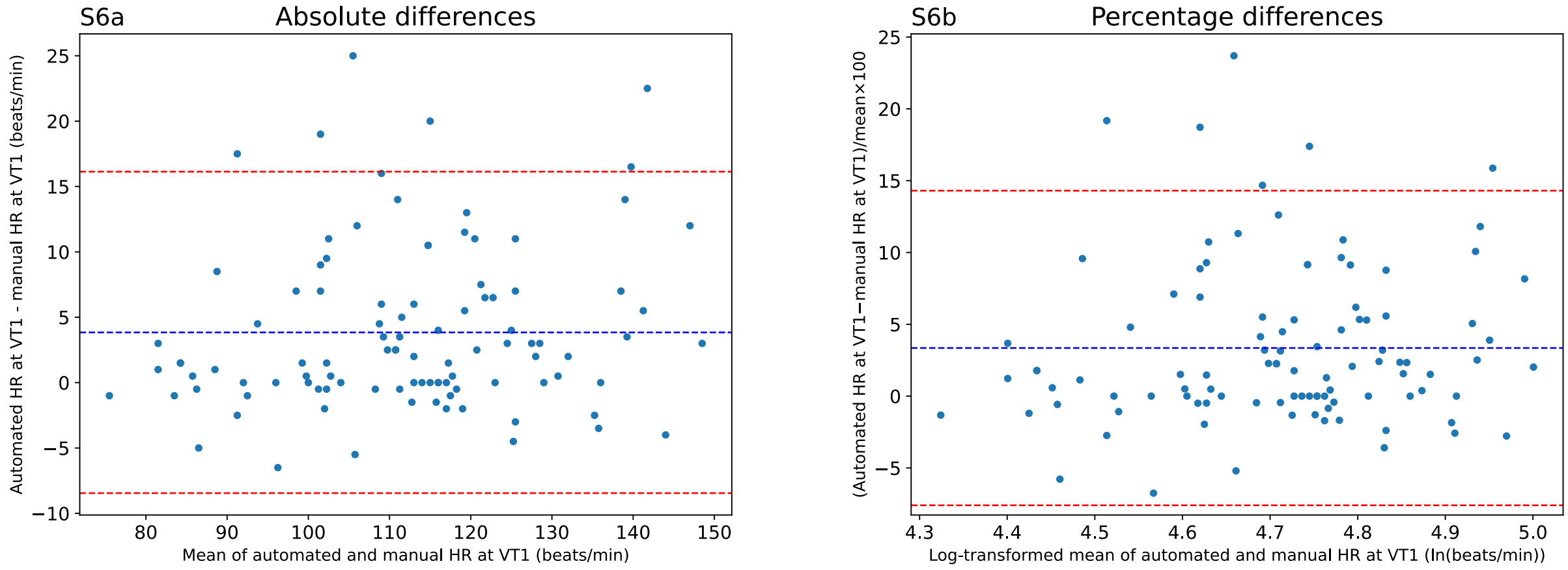

Fig. S6 (a) Absolute differences between methods. The mean bias was 3.8 beats/min (blue dashed line), with 95% LoA ranging from -8.5 to 16.3 (red dashed lines). Neither proportional bias (slope = 0.06, p = 0.116) nor heteroscedasticity was present (slope of absolute residuals = 0.04, p = 0.178). **(b)** Percentage differences between methods. The mean percentage difference was 3.4% ± 5.6% (blue dashed line), with 95% LoA ranging from −7.6 to +14.3% (red dashed lines). Neither proportional bias (slope = 3.4, p = 0.369) nor heteroscedasticity (slope = -0.50, p = 0.870) was present. Abbreviations: HR, heart rate; VT1, first ventilatory threshold.

Figure S7: Bland-Altman Plot for HR at VT1 in the female cohort (n = 160)

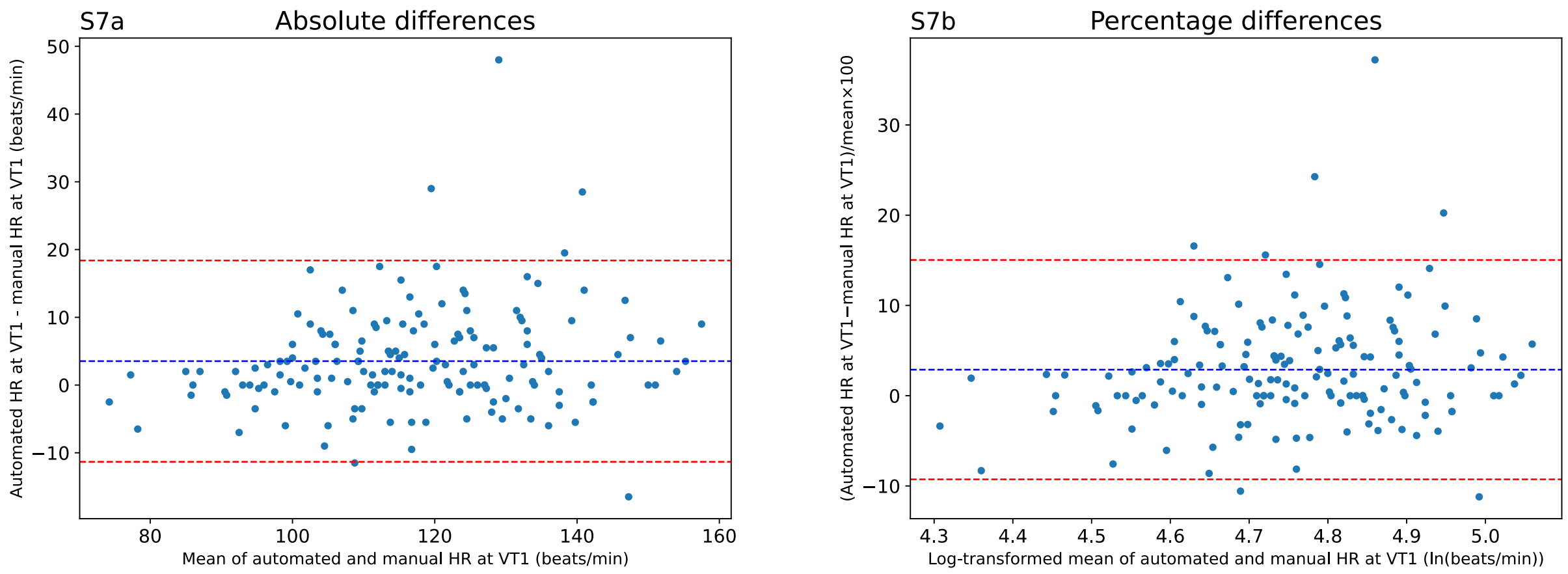

Fig. S7 (a) Absolute differences between methods. The mean bias was 3.5 beats/min (blue dashed line), with 95% LoA ranging from -11.3 to 18.4 (red dashed lines). Both proportional bias (slope = 0.07, p = 0.037) and heteroscedasticity were present (slope of absolute residuals = 0.09, p = 0.005). **(b)** Percentage differences between methods. The mean percentage difference was 2.9% ± 6.2% (blue dashed line), with 95% LoA ranging from −9.3 to +15.0% (red dashed lines). Neither proportional bias (slope = 6.4, p = 0.054) nor heteroscedasticity (slope = 4.47, p = 0.117) was present. Abbreviations: HR, heart rate; VT1, first ventilatory threshold.

Figure S8: Deming regression for HR at VT1 in overall cohort (n = 261)

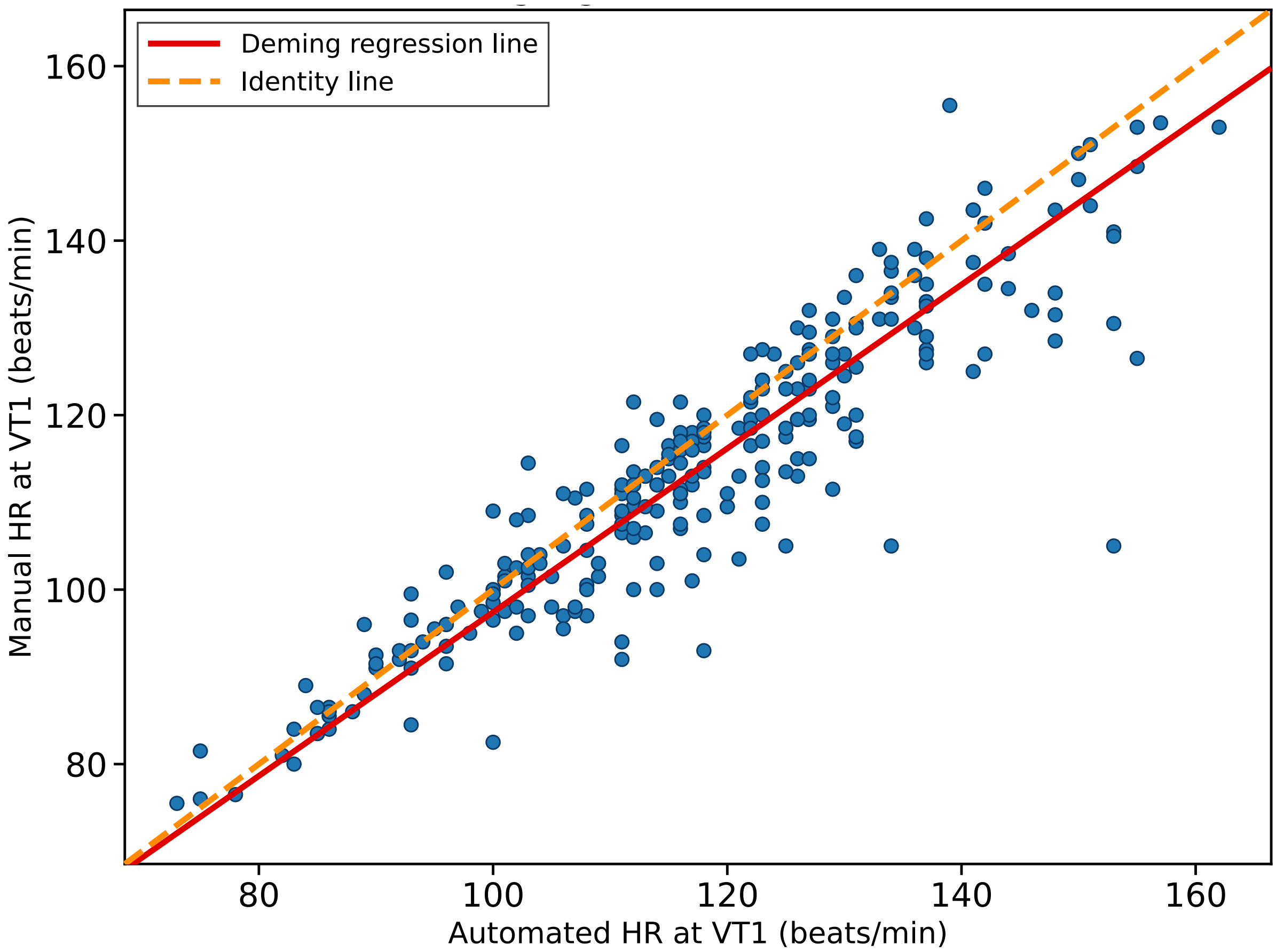

Fig. S8 Scatter plot illustrating the relationship between automated and manual HR at VT1 in the overall cohort. The red solid line represents the Deming regression line (slope 0.94, 95% CI 0.90–0.98; intercept 3.6 beats/min, 95% CI -1.6 to 8.4); the orange dashed line represents the identity line. Each dot represents one subject. Abbreviations: HR, heart rate: VT1; first ventilatory threshold.

Figure S9: Deming regression for HR at VT1 in male cohort (n = 101)

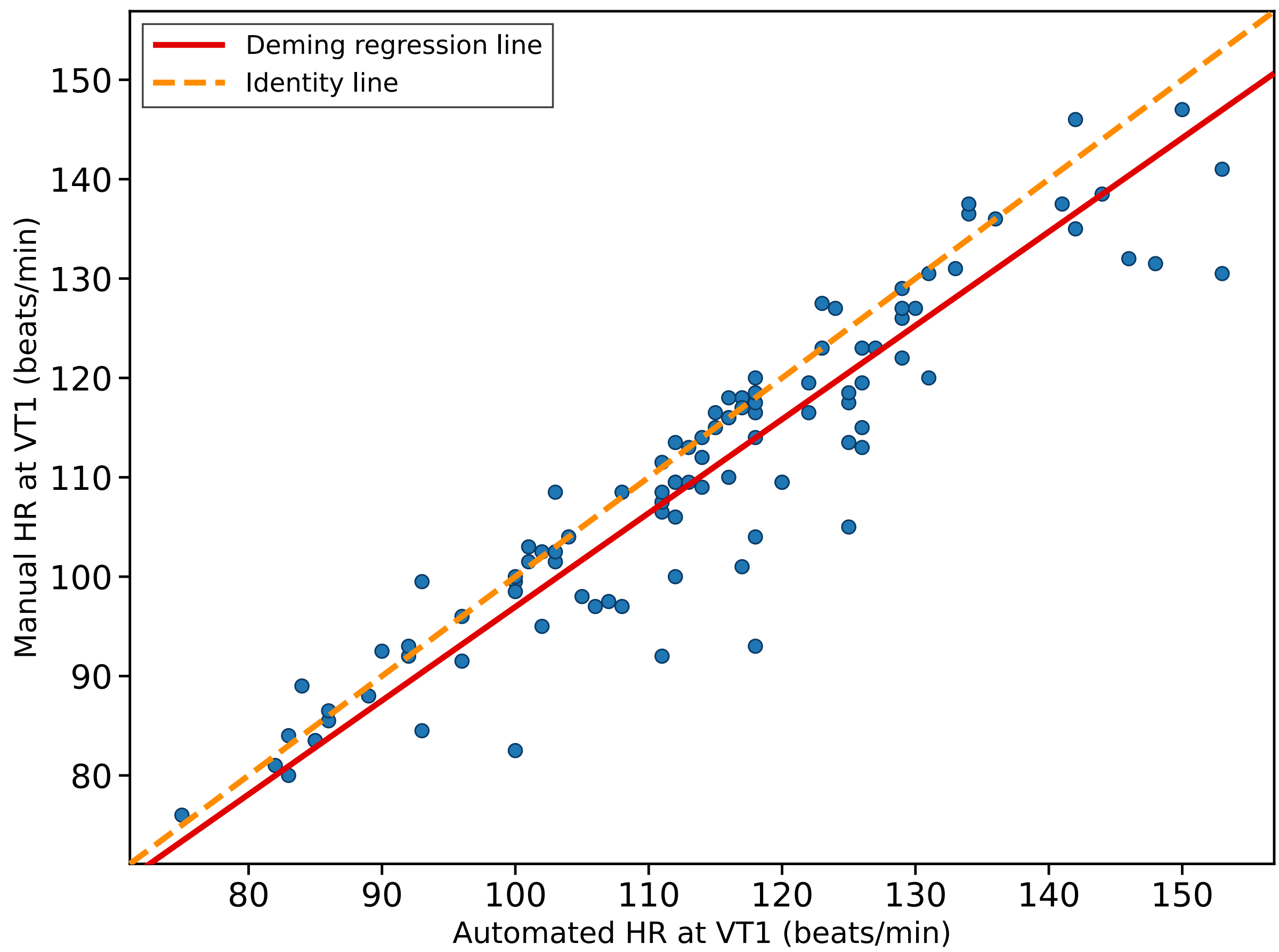

Fig. S9 Scatter plot illustrating the relationship between automated and manual HR at VT1 in the male cohort. The red solid line represents the Deming regression line (slope 0.94, 95% CI 0.88–1.01; intercept 2.6 beats/min, 95% CI -5.6 to 10.0); the orange dashed line represents the identity line. Each dot represents one subject. Abbreviations: HR, heart rate: VT1; first ventilatory threshold.

Figure S10: Deming regression for HR at VT1 in the female cohort (n = 160)

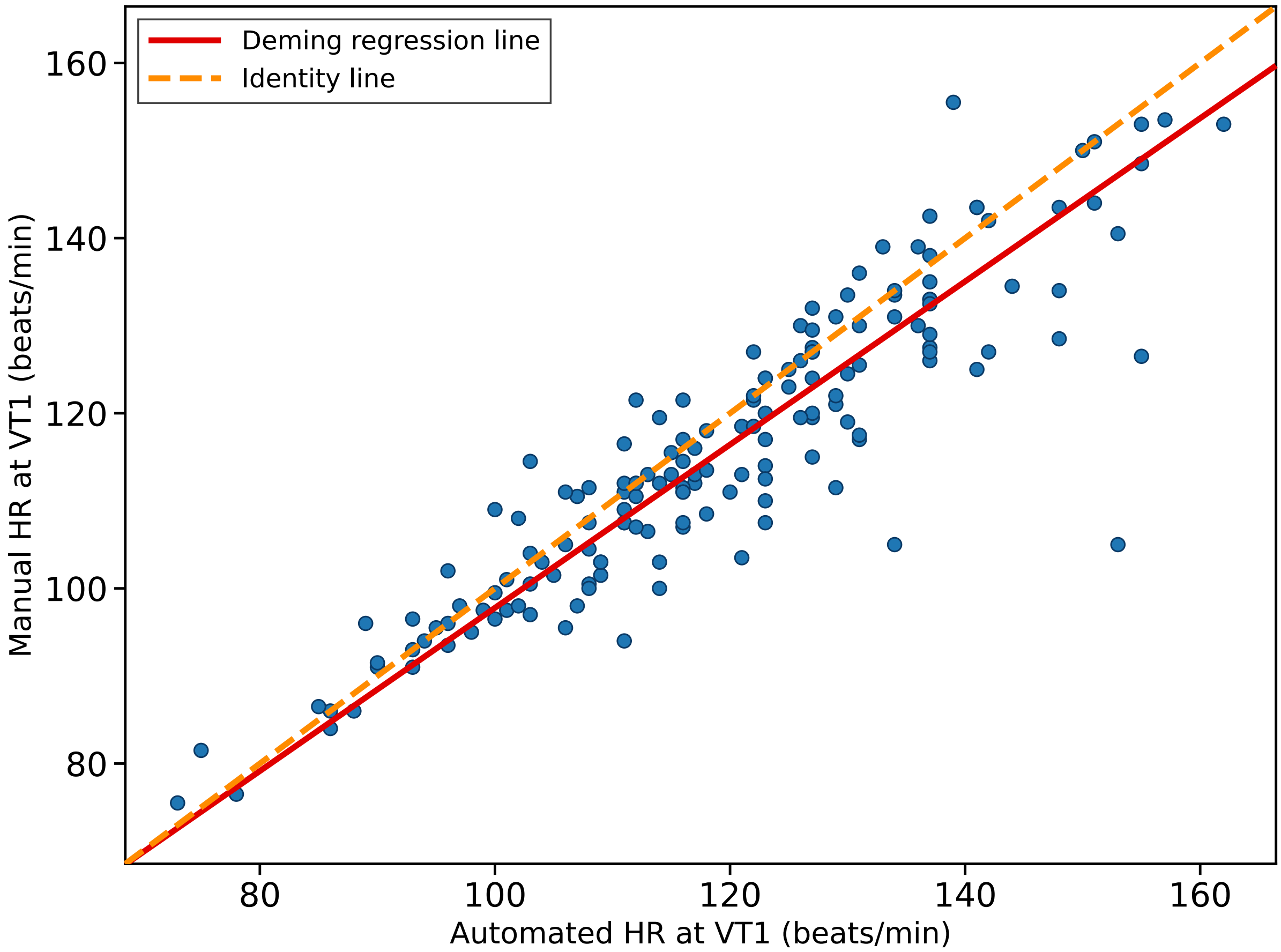

Fig. S10 Scatter plot illustrating the relationship between automated and manual HR at VT1 in the female cohort. The red solid line represents the Deming regression line (slope 0.93, 95% CI 0.88–0.99; intercept 4.6 beats/min, 95% CI -1.9 to 10.6); the orange dashed line represents the identity line. Each dot represents one subject. Abbreviations: HR, heart rate: VT1; first ventilatory threshold.

Figure S11: VCO_2–VO_2 curves of outlier cases according to Bland Altman analysis of absolute differences

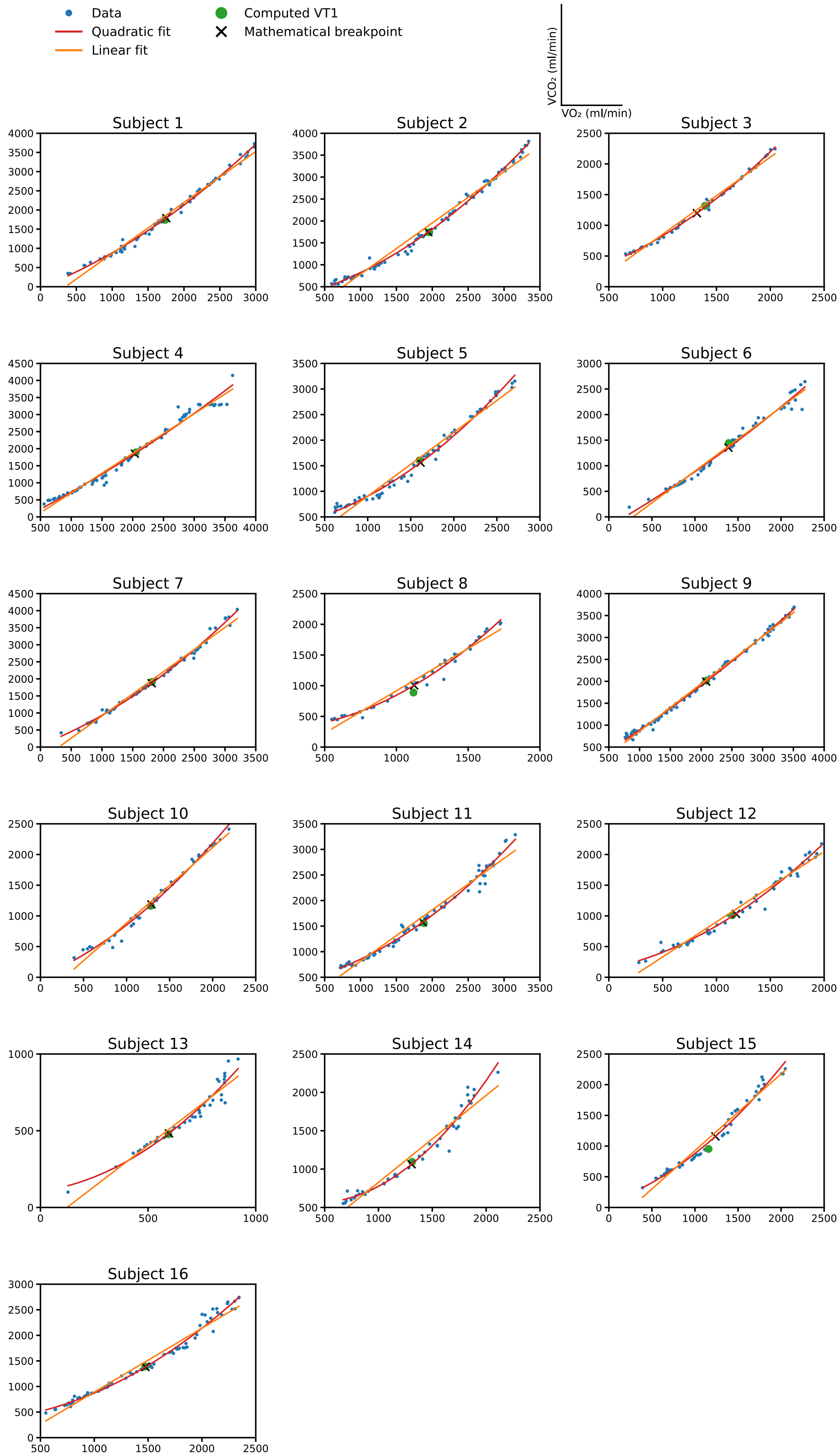

Fig. S11 Each panel displays the 10s averaged data (blue circles), their linear (orange line) and quadratic regression line (red line) and the automated (green circle), and manually assessed VT1 (red circle). Outliers reflect several curve morphologies: near-linear trajectories without a clear breakpoint (subjects 2, 5, 7, 13, 14, 15, 16), large VO_2 increments near the threshold reducing detection accuracy (subjects 1, 2, 3, 5, 7, 8, 10), gradual transitions without sharp inflection (subjects 4, 6, 8, 9, 11, 12) , where the manual method identifies the first deviation from linearity while the automated algorithm selects the point of maximum curvature, artefactual measurements biasing regression fitting (subject 3) and an early VT1 shifting the computed breakpoint rightward due to influence of many distal data points (subject 4). Curve 13 illustrates how a single measurement can strongly influence the quadratic regression, whereas visual assessment would likely have filtered this out.

Figure S12: VCO_2–VO_2 curves of outlier cases according to Bland Altman analysis of percentage differences

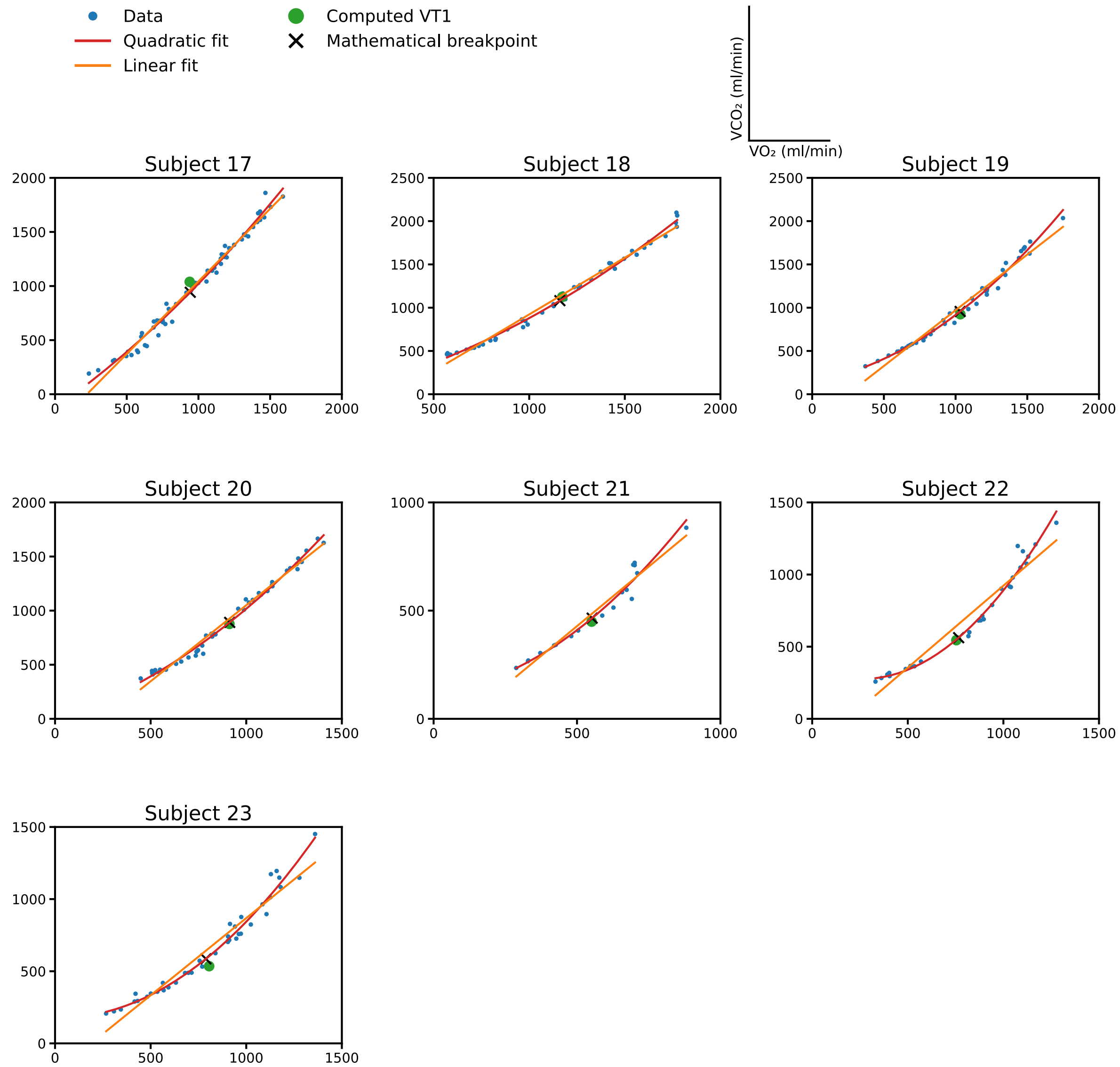

Fig. S12 Each panel displays the 10s averaged data (blue circles), their linear (orange line) and quadratic regression line (red line) and the automated (green circle), and manually assessed VT1 (red circle). Subjects 3,4, 6, 9-16 were also identified as outlier cases in the Bland–Altman analysis of absolute differences and are represented in Figure S11. Recurring morphological patterns include curves with large VO_2 increments near the expected threshold (subjects 3 and 10), gradual transitions without a sharp inflection rendering visual estimation inherently subjective (subjects 4, 6, 9, 11-17, 21), and cases in which the automated algorithm selected a later breakpoint than the manual method identifying the first deviation from linearity (subjects). As expected, strongly linear curves in this analysis (subjects 17, 18, 20) are associated with relatively low VO_2 values, in contrast to those identified in the Bland–Altman analysis of absolute differences

References

1. Cheng B, Kuipers H, Snyder AC, Keizer HA, Jeukendrup A, Hesselink M. A new approach for the determination of ventilatory and lactate thresholds. Int J Sports Med. 1992;13(7):518-22.
